# Asparagine, colorectal cancer, and the role of sex, genes, microbes, and diet: A narrative review

**DOI:** 10.3389/fmolb.2022.958666

**Published:** 2022-08-25

**Authors:** Xinyi Shen, Abhishek Jain, Oladimeji Aladelokun, Hong Yan, Austin Gilbride, Leah M. Ferrucci, Lingeng Lu, Sajid A. Khan, Caroline H. Johnson

**Affiliations:** ^1^ Department of Chronic Disease Epidemiology, Yale School of Public Health, Yale University, New Haven, CT, United States; ^2^ Department of Environmental Health Sciences, Yale School of Public Health, Yale University, New Haven, CT, United States; ^3^ Division of Surgical Oncology, Department of Surgery, Yale University School of Medicine, New Haven, CT, United States

**Keywords:** asparagine, asparagine synthetase (ASNS), asparaginase, colorectal cancer (CRC), sex difference, diet, microbes

## Abstract

Asparagine (Asn) and enzymes that catalyze the metabolism of Asn have been linked to the regulation and propagation of colorectal cancer (CRC). Increased Asn and asparagine synthetase (ASNS) expression, both contribute to CRC progression and metastasis. In contradistinction, L-asparaginase (ASNase) which breaks down Asn, exhibits an anti-tumor effect. Metabolic pathways such as KRAS/PI3K/AKT/mTORC1 signaling and high SOX12 expression can positively regulate endogenous Asn production. Conversely, the tumor suppressor, TP53, negatively impacts ASNS, thus limiting Asn synthesis and reducing tumor burden. Asn abundance can be altered by factors extrinsic to the cancer cell such as diet, the microbiome, and therapeutic use of ASNase. Recent studies have shown that sex-related factors can also influence the regulation of Asn, and high Asn production results in poorer prognosis for female CRC patients but not males. In this narrative review, we critically review studies that have examined endogenous and exogenous modulators of Asn bioavailability and summarize the key metabolic networks that regulate Asn metabolism. We also provide new hypotheses regarding sex-related influences on Asn, including the involvement of the sex-steroid hormone estrogen and estrogen receptors. Further, we hypothesize that sex-specific factors that influence Asn metabolism can influence clinical outcomes in CRC patients.

## 1 Introduction

Globally, colorectal cancer (CRC) is the third most commonly diagnosed cancer and the second leading cause of cancer-related deaths (American Society of Clinical Oncology, 2022), posing a major global public health issue. CRC incidence and mortality rates are higher in male patients compared to female patients (American Cancer Society, 2020); however, these rates vary by anatomic location of the tumor (Weiss et al., 2011), with right-sided tumors having a poorer prognosis (Gervaz et al., 2016) and occurring more frequently in females (Kim et al., 2015). There is also high interindividual variation in terms of treatment strategies and prognosis due to differences in mutational burden (Huyghe et al., 2019), metabolism (Zhang et al., 2020), and age of the patient (van Eeghen et al., 2015).

Historically, CRC risk assessment has mainly focused on genetic susceptibility (Hereditary Nonpolyposis Colorectal Cancer (HNPCC), Familial Adenomatous Polyposis (FAP)), and lifestyle factors (e.g., obesity, diet, physical inactivity, and smoking) (Johnson et al., 2013). More recently, there has been a plethora of studies that have explored the use of -omics technologies to identify novel diagnostic and prognostic biomarkers to guide CRC clinical care (Dalal et al., 2020). Of these technologies, metabolomics holds great promise as it allows for the comprehensive and simultaneous examination of both exposures and their biological effects (through examination of disruption to metabolic pathways). This enables for a deeper understanding of the complex etiology of exposures in cancer, as well as the gene-environmental continuum. Further, considering that most cases are sporadic, and caused due to an exposure or set of exposures interacting within a particular genetic landscape, there are relevant clinical implications (Rattray et al., 2017).

Our recent publication, which used metabolomics data to identify prognostic biomarkers of CRC, revealed a unique association between enhanced asparagine (Asn) abundance in CRC tumors and poorer survival in female patients, an effect which was not seen in male patients (Shen et al., 2022). Furthermore, exploring cancer genomics database showed that elevated expression of asparagine synthetase (ASNS) correlated with poorer clinical outcomes in female CRC patients (Cai et al., 2020).

Asn has been previously linked to other cancer types and targeted for treatment. In the 1970s, asparaginase (ASNase), an enzyme extracted from *Escherichia coli* (*E. coli*) was found to decrease Asn levels by catalyzing the hydrolysis of Asn, leading to the clinical application of L-asparaginase as an anti-cancer therapy for acute lymphoblastic leukemia (ALL) patients (Clarkson et al., 1970). ASNase produced by *Streptomyces fradiae* NEAE-82 has cytotoxicity, and anti-cancer effects in human epithelial colorectal adenocarcinoma cells (Caco-2 cells), manifesting its potential in treatment of CRC (El-Naggar et al., 2016). An *in vitro* study involving Asn concentration in cell lines of other cancer types revealed that endogenous Asn production by ASNS may promote protein and nucleotide synthesis, thereby contributing to increased cell proliferation (Krall et al., 2016). In models of mutant *KRAS* CRC, ASNS expression is upregulated by the KRAS-activated signaling pathway, which increases levels of Asn and decreases levels of aspartate (Asp, the metabolic substrate for ASNs) (Toda et al., 2016). This indicates that *ASNS* might be a novel therapeutic target for mutant *KRAS* CRC given its link to increased ASNS expression, Asn generation and propagation of tumor growth. However, as yet, ASNS has not been targeted in any clinical trials.

Another important transcription factor, SRY-Box Transcription Factor 12 (SOX12), was shown to transactivate *ASNS* expression. In addition, downregulation of ASNS, inhibited SOX12-mediated CRC cell proliferation and metastasis in tumor cells overexpressing SOX12 (Du et al., 2019). Intracellular Asn promotes activation of the mammalian target of rapamycin complex 1 (mTORC1) by serving as an exchange factor to regulate uptake of extracellular amino acids such as serine, arginine, and histidine which are required for mTORC1 signaling; such a modulation of mTORC1 by Asn also drives protein and nucleotide synthesis in cancer cells (Krall et al., 2016). mTORC1 activation also promotes tumorigenesis by enhancing translation of pro-tumorigenic genes, inhibiting autophagy, increasing angiogenesis, and upregulating lipid levels (Zoncu et al., 2011).

Most studies exploring the role of Asn, ASNS and ASNase in CRC have not examined sex-specific effects. Thus, research gaps exist in understanding the mechanisms of sex-specific Asn metabolism in CRC. Additionally, it is crucial to obtain a deeper understanding of the biological sources of Asn and how they influence CRC tumor proliferation through metabolic regulation.

In this narrative review, we summarize advances in knowledge regarding the biological sources of Asn, and how Asn could affect CRC survival, proliferation, and metastasis. We also investigate potential ways to regulate extracellular Asn bioavailability through diet and microbial metabolism. We also hypothesize how sex-related factors could play a role in the regulation of Asn production and function.

## 2 Intracellular asparagine production and colorectal cancer

In this section, we discuss intracellular *de novo* synthesis of Asn, which is catalyzed by ASNS. Importantly, ASNS is regulated upstream by KRAS, SOX12 and p53, and we describe how mutations to these genes can affect intracellular Asn levels. We then discuss how intracellular Asn levels can be increased through proteasomal recycling of proteins (Davidsen and Sullivan, 2020).

### 2.1 *De novo* synthesis of asparagine

Within the cell, ASNS catalyzes the synthesis of Asn in an ATP-dependent reaction, using glutamine (Gln) and Asp as substrates. This intracellular production of Asn is somewhat dependent on the functioning of the mitochondrial electron transport chain (ETC) as inhibition of the ETC using metformin impedes the conversion of Asp from Gln, and therefore affects *de novo* synthesis of Asn (Davidsen and Sullivan, 2020; Krall et al., 2021). ASNS-catalyzed synthesis of Asn is also dependent on somatic mutation patterns within the cancer cells, since *ASNS* expression is regulated by *KRAS, SOX12* and *TP53,* which we discuss further here.

#### 2.1.1 KRAS

Somatic mutations to the *KRAS* gene are frequently found in colon adenocarcinomas (Bos, 1989), and are associated with poor prognosis and drug resistance (Meng et al., 2021). One of the mechanisms in which mutant KRAS contributes to tumor progression is through activation of the phosphoinositide 3-kinase (PI3K) /protein kinase B (AKT)/mTOR pathway by binding to the p110α subunit of PI3K, causing a procarcinogenic signaling cascade (Hua et al., 2019). Downstream of KRAS, activating transcription factor 4 (ATF4) and nuclear factor erythroid 2-related factor 2 (NRF2) can upregulate *ASNS* expression (Krall et al., 2021), inducing apoptotic suppression and mTORC1 activation (Gwinn et al., 2018). Additionally, macropinocytosis, a process that engulfs extracellular content into vesicles, is correlated to Asn synthesis and is elevated to assist mutant *KRAS* cells to adapt to nutrient depletion. PI3K is involved in the activation of micropinocytosis (Hanada et al., 2021). Moreover, impeding macropinocytosis and Asn depletion resulted in a prominent tumor growth suppression effect in mutant *KRAS* CRC cells *in vivo* (Hanada et al., 2021).

Studies analyzing mutant *KRAS* CRC found that solute carrier family 25 member 22 (SLC25A22) promoted proliferation and migration of human CRC tumor cells which also resulted in the developments of metastases in CRC xenograft mouse models (Wong et al., 2016). Additionally, SLC25A22 depletion inhibits Asp-derived Asn synthesis by lowering Asp levels which suppresses proliferation of *KRAS* mutant CRC cells (Wong et al., 2016). In another study, SLC25A22 was shown to serve as an essential metabolic regulator for CRC progression by promoting the synthesis of Asn in mutant KRAS CRC cells (Li et al., 2017).

#### 2.1.2 TP53

Tumor protein (p53) is a tumor suppressor encoded by the *TP53* gene (Takayama et al., 2006) and is mutated in ∼60% of CRC cases (Nakayama and Oshima, 2019). Deng et al. discovered that the anti-tumoral activity of p53 acts by downregulating ASNS expression to limit Asn synthesis in CRC, interrupting Asn-Asp equilibrium *in vivo* and *in vitro* (Deng et al., 2020). The authors devised a model that illustrated a reciprocal regulation of p53 and ASNS through LKB1-AMPK signaling: when Asn synthesis is inhibited due to transcriptional downregulation of ASNS expression by p53, Asp will accumulate to activate LKB1-AMPK signaling as indicated by increased LKB1-mediated AMPK phosphorylation, causing p53-dependent cell cycle arrest. In the absence of p53, Asn inhibits LKB1 activity and halts this p53-dependent cell cycle arrest to promote tumor cell proliferation and survival (Deng et al., 2020).

#### 2.1.3 SOX12

*SOX12*, one of the sex-determining region Y-associated high-mobility group (HMG) cassette (*SOX*) family genes, and its overexpression has been associated with the promotion of tumor progression (Huang et al., 2015) and poor prognosis in CRC (Du et al., 2019). *SOX12* facilitates Asn synthesis by transactivating glutaminase (GLS), glutamic oxaloacetic transaminase 2 (GOT2), and ASNS(Du et al., 2019). Of note, GLS is an amidohydrolase enzyme that catalyzes the production of glutamate (Glu) from Gln and is essential for *de novo* Asn synthesis (Sugimoto et al., 2015). Furthermore, GOT2 converts oxaloacetate to Asp (Feist et al., 2018), a substrate for Asn synthesis.

### 2.2 Proteasomal recycling of asparagine, and asparaginase resistance

The proteasome is essential in tumor cells for maintaining the decomposition of intracellular proteins such as p53 that aids DNA repair, and p21 and p27 that impede cancer cell growth, resulting in the promotion of tumor cell survival and proliferation (Almond and Cohen, 2002). Consequently, proteasomal inhibition is a major therapeutic target for cancer chemotherapy (Almond and Cohen, 2002). However, proteasome inhibitors are typically accompanied by resistance, which could be addressed by targeting upstream regulators (Manasanch and Orlowski, 2017). One example of this is restricting the actions of the upstream regulator glycogen synthase kinase 3 (GSK3). GSK3-dependent protein ubiquitination and consequent destruction at the proteasome can generate sources of free Asn. The actions of GSK3 can be restricted by WNT-dependent stabilization of proteins (WNT/STOP) to mitigate resistance to ASNase in CRC (Hinze et al., 2020). When WNT/STOP is off, GSK3-dependent protein degradation remains unperturbed and produces catabolic free Asn which induces ASNase resistance in CRC; whereas CRCs with R-spondin (RSPO) fusions such as RSPO3 have activated WNT/STOP which inhibits the proteasome, limiting Asn availability and thus increasing vulnerability to ASNase (Hinze et al., 2020). Moreover, tumor cells that have *KRAS/TP53* mutations, are forced to survive on Asn recycled from protein degradation in the proteasome as autophagy is inhibited and not available as an alternative protein recycling pathway (Hinze et al., 2020).

As described above, tumor cells are sensitized to Asn depletion through actions of ASNase which subsequently increases Asn levels through proteasomal release (Davidsen and Sullivan, 2020). Under normal cellular quiescent conditions, WNT signaling is silenced through proteasomal degradation of β-catenin mediated by the APC/Axin/GSK-3 destruction complex (Fearon, 2011). However, tumor formation, mediated by elevated WNT signaling leads to the accumulation of cytosolic and nuclear β-catenin which promotes cell proliferation. WNT activity, therefore, prevents proteasomal degradation, an event that leads to the recycling of protein into free Asn and consequently survival of tumor cells (Davidsen and Sullivan, 2020).

Therefore, proteasomal degradation that recycles protein into Asn is associated with ASNase resistance. Activation of WNT/STOP signaling may improve the therapeutic effect of ASNase in CRC.

## 3 Extracellular sources of asparagine

**FIGURE 1:**
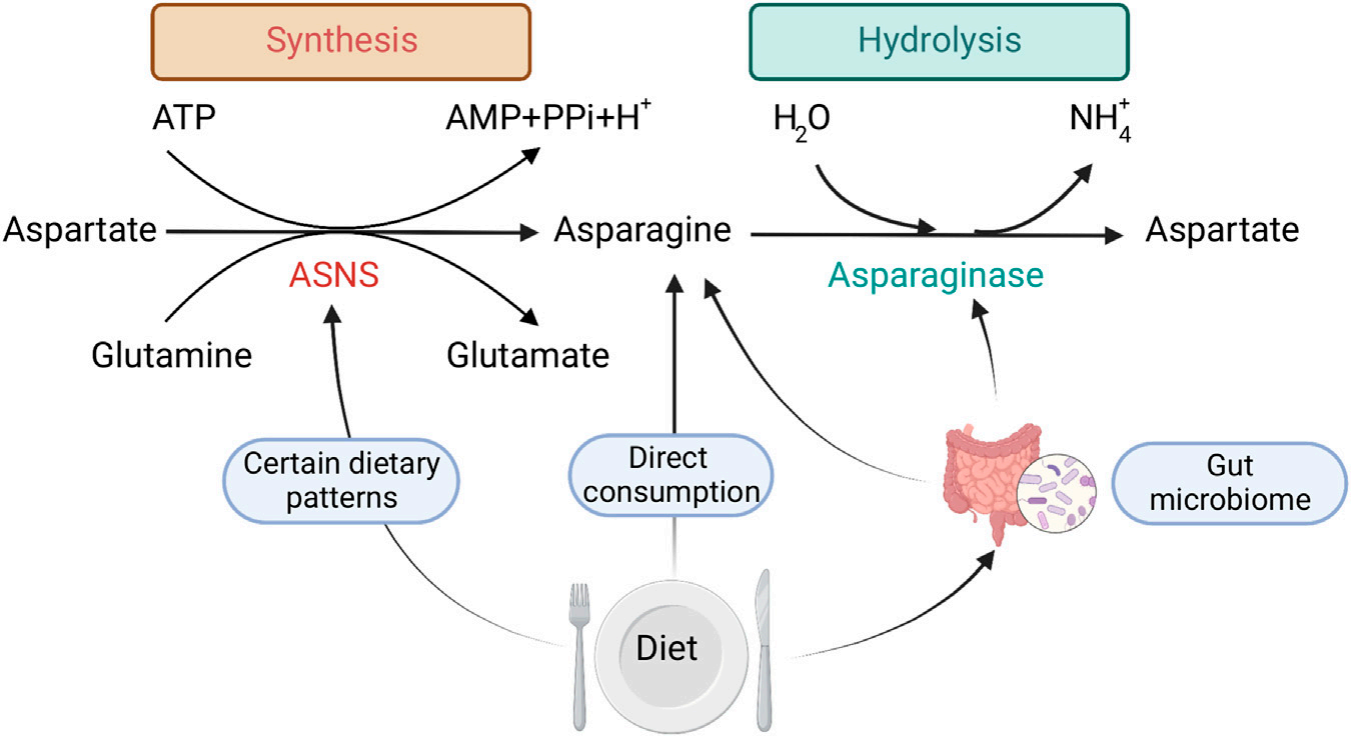
Mechanisms by which dietary exposure changes Asn bioavailability: 1) direct consumption of Asn, 2) dietary patterns that regulate ASNS expression, 3) gut microbial metabolism. Created with BioRender.com.

In this section, we describe the different biological sources of Asn that can result in increased extracellular levels of Asn. We show that extracellular Asn can be generated through the ingestion of dietary components and can be hydrolyzed by the actions of microbial ASNases (Figure 1). Extracellular Asn is important to quantify, as it can be transported into cancer cells by SLC1A5 and aid in tumor growth. Of note, SLC1A5 is a promising therapeutic target for restricting cancer progression (Scalise et al., 2018). We also indicate potential directions in future studies that can minimize gaps in our current knowledge of this area.

### 3.1 Dietary exposure

Dietary intake of foods high in Asn, and dietary patterns that might influence *ASNS* expression mediate free Asn levels. In this context, other amino acids that are involved in Asn synthesis and are acquired exogenously may also serve as indirect regulators of Asn bioavailability. However, it is difficult to investigate the exact dietary sources and consumption of Asn (and other amino acids), due to the lack of sufficient and reliable nutrient databases that quantify micronutrients such as Asn in foods. Most existing nutrient databases do not provide information specifically on Asn (Lenders et al., 2009). This may be due to the difficultly in assessing levels of Asn in foods *via* common laboratory methods. Since most of the common methods of assessing dietary intake in populations, such as food frequency questionnaires (FFQs), rely on nutrient databases, most large epidemiologic studies cannot quantify Asn levels within the diet. Therefore, to our knowledge, the direct association between self-reported dietary intake of Asn and CRC has not been studied.

An alternative approach would be to examine Asn abundance in foods that are observed to be associated with the risk of CRC. For example, the consumption of red meat is associated with a higher risk of CRC development (Thanikachalam and Khan, 2019), and short-term red meat consumption is positively associated with higher serum levels of Asn (Guan et al., 2021), however, data on Asn levels in red meat compared to other foods is not available. Even though Asn may be higher in the serum of those consuming red meat, the role of dietary Asn from red meat in CRC risk would be difficult to tease apart as there are other components of red meat that are linked to CRC development such as N-nitroso compounds, and those that are formed when red meat is cooked at high temperatures such as heterocyclic amines and polycyclic aromatic hydrocarbons (Aykan, 2015). It is still too premature to conclude a link between red meat, Asn levels, and CRC, especially since vegetables which are often associated with reduced risk of CRC are also sources of Asn and would thus confound these associations.

Food additives could also be linked to cancer. A recent study hypothesized that consumption of animals fed with ractopamine might be pro-tumoral for CRC patients as ractopamine could increase ASNS expression in the skeletal muscle of pigs (Fan, 2022).

Some dietary patterns, such as the vegan diet (not consuming any animal products), and vegetarian diets (not consuming any meat or fish) (Clarys et al., 2014), have been shown to increase levels of intestinal ASNase (assessed in fecal samples) in individuals consuming these diets as reported *via* FFQs (De Angelis et al., 2020). Whereas other diets which have dietary protein restriction, or an imbalanced dietary amino acid composition have shown that the *ASNS* gene can be activated through amino acid response (AAR) that limits single essential amino acids (Balasubramanian et al., 2013).

The consumption of specific dietary components has been shown to modulate proliferation, gene expression, and metabolism of gut microorganisms, leading to changes in protein synthesis (Figure 1), which could further affect Asn levels. For example, dietary consumption of sourdough bread might bring a benefit to host health by increasing healthy gut microorganisms in the colon (Da Ros et al., 2021), such as boosting the growth of *Bacteroides* that express ASNase (Abbondio et al., 2019).

Overall, with regards to dietary sources of Asn, laboratory studies that accurately assess Asn levels in foods are needed, so that Asn can be accurately added to nutrient databases. If dietary Asn could be accurately quantified, then associations between dietary Asn and CRC risk could be evaluated in existing and future large epidemiologic populations.

Experimental models have shown that limiting dietary intake of Asn can decrease cancer growth. A study found that decreasing Asn in chow that was fed to mice with a breast cancer xenograft had a protective effect on metastatic cancer progression. Furthermore, enhancing Asn in the diet resulted in more invasive tumors (Knott et al., 2018). Dietary restriction to exogenous non-essential amino acids, including Asn, also hindered the growth of lung cancers that harbored Kelch-like ECH-associated protein 1 (KEAP1)/NRF2 mutations *in vivo*, wherein the oxidative stress response was activated (LeBoeuf et al., 2020). In a wider variety of cancer types, inhibition of the ETC was shown to impair mitochondrial respiration by reducing ATP and Asp levels, thus limiting Asn synthesis. With ETC impairment, sufficient exogenous Asn supply rescued ATF4 protein levels and ASNS expression, and restored mTORC1 activation; restricted Asn dietary intake together with ETC inhibition further suppressed proliferation (Krall et al., 2021).

### 3.2 Metabolism of asparagine by the microbiome

There are trillions of microbiota residing in the human gut that play a critical role in health (Lozupone et al., 2012). These microbes are known to aid in the production of metabolites including short-chain fatty acids, vitamins, indoles and amino acids that ultimately impact the host metabolism (Sharon et al., 2014). The gut microbiome has been associated with cancer progression, and alterations in the diversity of gut microbiome can also affect the course of the treatment (Gopalakrishnan et al., 2018). The gut microbiome has been shown to mediate ASNS and ASNase activity which can alter Asn levels, and could also ultimately impact the efficacy of L-ASNase therapy (Amer et al., 2013; Hillman et al., 2020; Dunn et al., 2021). The efficacy of ASNase treatment is determined by its ability to maintain the depleted levels of Asn for a prolonged period of time (Pieters et al., 2011; Covini et al., 2012). Dunn et al. reported that pediatric ALL patients treated with ASNase therapy showed distinctive gut microbial composition that correlated with serum ASNase activity (Dunn et al., 2021). Patients with decreased serum ASNase activity had enrichment of *Escherichia*, whereas those with increased serum ASNase activity has an enrichment of *Bacteroides* and *Streptococcus* (Dunn et al., 2021).

Microbial species express the *ASNS* gene and two ASNases, *ansA* and *ansB*. Therefore, the differential taxonomic distribution of these genes could underlie the major reasons for such kind of microbial community distinction based on increased or decreased serum ASNase activity. For example, it had been found that *E. coli* contains all three genes in its genome, *Blautia [Ruminococcus] gnavus* and *Bacteroides vulgatus* contains *ASNS* and *ansB*, while *Bacteroides ovatus*, *Bacteroides stercoris*, *Bacteroides uniformis*, *Faecalibacterium prausnitzii*, *Roseburia intestinalis* contains *ASNS* and *ansA* (Dunn et al., 2021). There are some microbial species that contain only one of these genes. For example, *Bifidobacterium longum*, *Collinsella aerofaciens* contains only *ansA*, *Blautia obeum*, *Blautia torques* contains only *ASNS*, while *Clostridium bartlettii, Veillonella parvula* contains *ansB* (Dunn et al., 2021). There has been a limited number of studies that showed the association between microbiome and ASNase treatment in cancer. Therefore, future studies to establish the predictive microbial biomarkers that could potentially impact ASNase levels during treatment are warranted. The manipulation of these microbial species and their associated genes (*ASNS*, *ansA* and *ansB*) could aid in optimizing the patient’s response to ASNase therapy.

Han et al. designed a systematic metabolomics-based pipeline for studying the functional microbiome and discovered that Asn and Gln can be used as sole nitrogen sources by various bacteria such as *Bacteroidetes* (Han et al., 2021). When grown on media supplemented with Asn, *Bacteroidetes* taxa growth was strongly correlated with an L-asparaginase II homologue (*ansB*; >59% identity), which was essential for growth when Asn was the only nitrogen source (Han et al., 2021). Using their data explorer pipeline, levels of Asn in feces and cecal contents was significantly lower in *Bacteroides thetaiotaomicron* (Bt.) inoculated mice compared to germ-free control (Han, 2020). It was shown that some microbes that were grown in mega media had decreased levels of Asn compared to media controls (i.e. with no bacteria), such as *E. coli and Bacteroides* species including *Bt.* and *Bacteroides caccae, Bifidobacterium dentium,* and *Blautia producta*., indicating that these bacteria used Asn as a growth source, and thus could affect Asn levels in the gut and perhaps of CRC patients. Whereas there are side effects associated with ASNase administration; *E. coli* ASNase can bind and inhibit the concanavalin A receptor, which accounts for non-immunological side effects and toxicities by ASNase (Van Trimpont et al., 2022). Moreover, the *Rhodospirillum rubrum* mutant L-ASNase is unique to other bacterial ASNases as it can penetrate cancer cells *via* a clathrin receptor-dependent manner that suppressed telomerase activity and led to asparagine-independent cytotoxicity (Zhdanov et al., 2017).

## 4 Sex-related influences on asparagine metabolism

**FIGURE 2:**
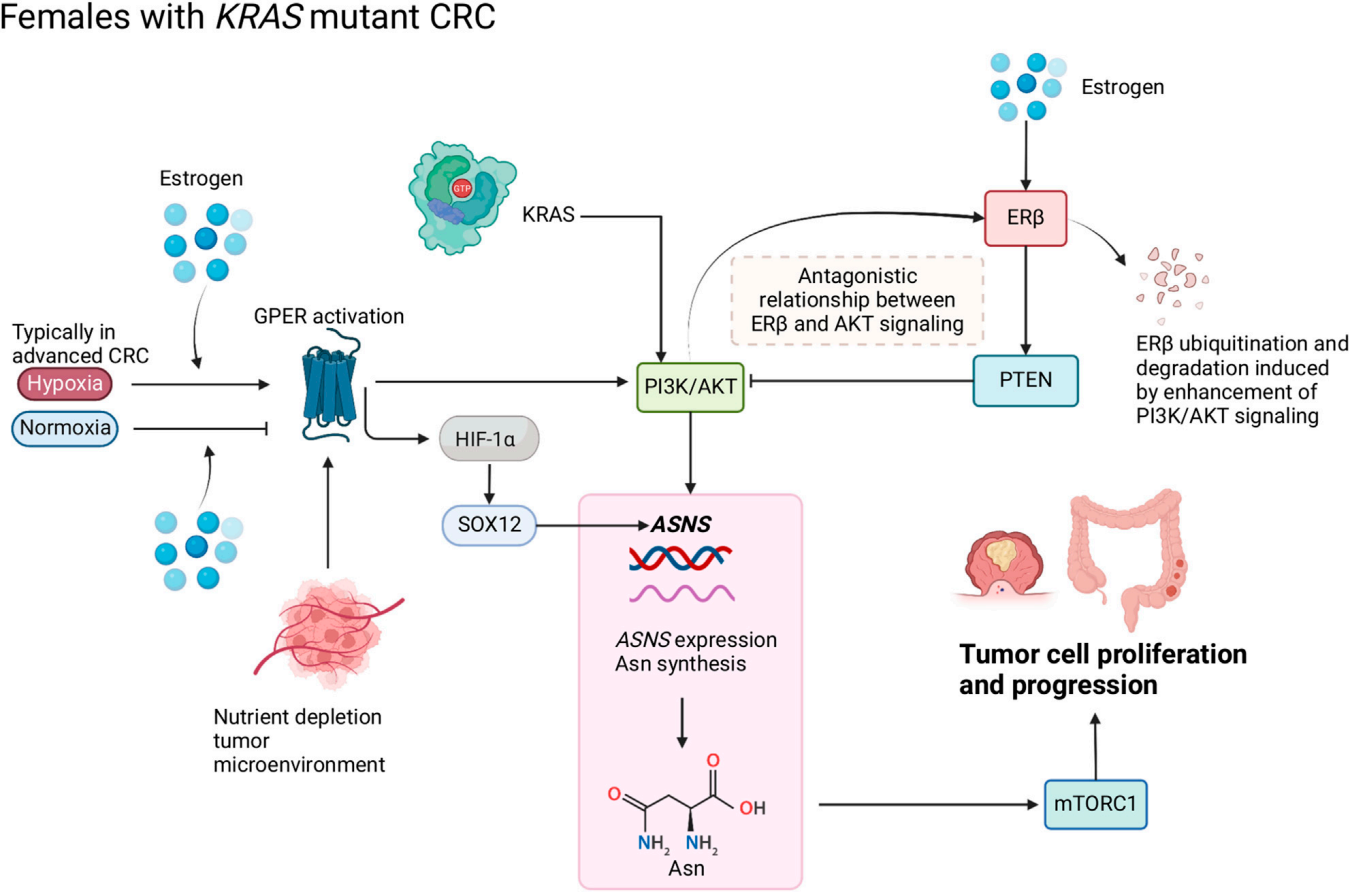
Schematic of Asn bioavailability and Asn metabolism in female patients with CRC. Estrogen regulates the antagonistic relationship between ERβ and AKT signaling. Oxygen levels modify the function of estrogen and GPER that target hypoxia-inducible factor (HIF)-1α and subsequently influence Asn synthesis through PI3K/AKT signaling. Created with BioRender.com.

**FIGURE 3:**
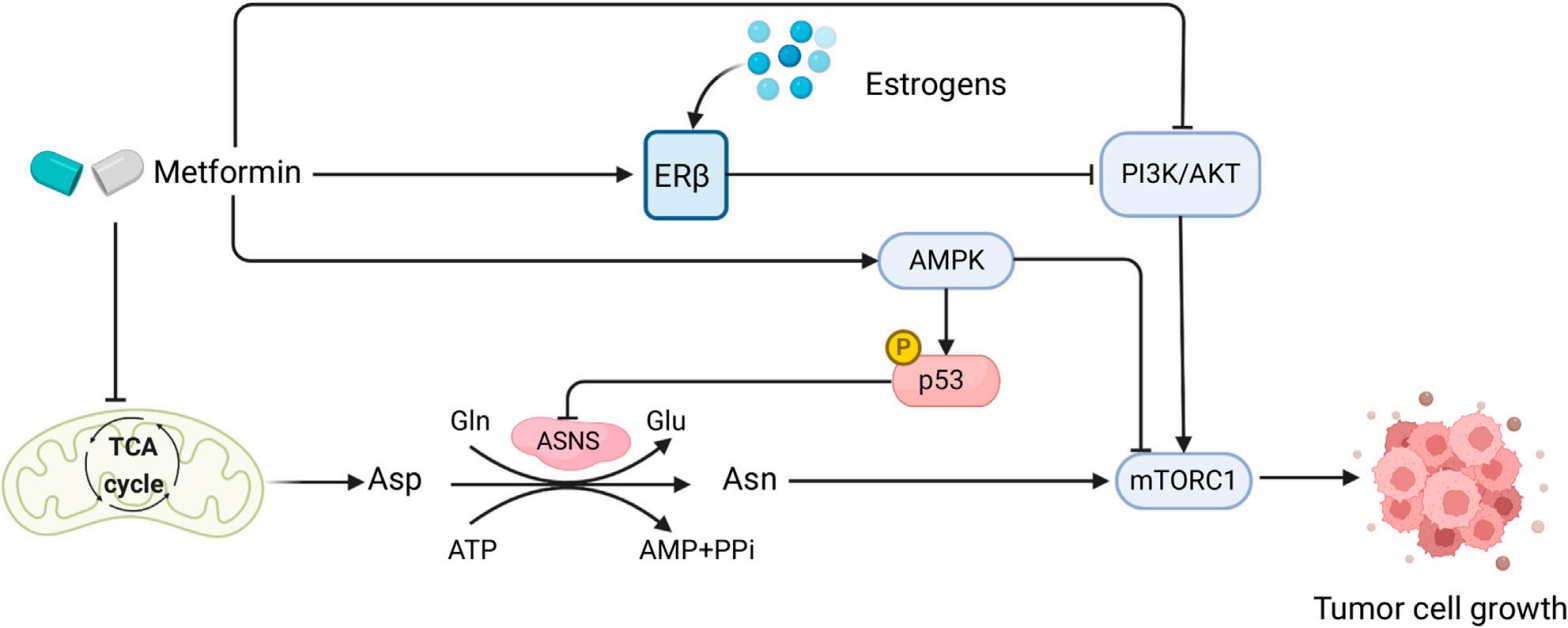
Sex interacts with the effect of metformin in CRC patients and mediates Asn metabolism. Created with BioRender.com.

In this section, we discuss the connection between Asn, CRC prognosis and sexual dimorphism. We examine sex-specific differences in ion channel activation and its links to proteasomal production of Asn. We also discuss sex-specific regulation of the PI3K/AKT pathway *via* phosphatase and tensin homologue (PTEN), and the role of the novel membrane bound G protein coupled estrogen receptor (GPER) (Figure 2) which is sensitive to nutrient supply and may also affect ASNS expression. Finally, we compare the similarities between pathway deregulation in diabetes mellitus and the involvement of AMPK in the sex-specific responses observed (Figure 3).

### 4.1 Sex-specific differences in ion channel activation link to the proteasome

As previously discussed, Asn can be generated through the breakdown of proteins in the proteasome which is regulated by β-catenin. Sex-specific differences in the expression of voltage-gated K+ (Kv) channels such as those in the KCNQ family (KCNQ1 and KCNE3) can affect this process; the sex-steroid hormone 17β-estradiol inhibits the KCNQ1:KCNE3 channel complex, resulting in a relatively higher proportion of free homomeric KCNQ1 that inhibits WNT/β-catenin signaling pathway (Abancens et al., 2020). Therefore, estrogens regulate Kv channel expression and might indirectly decrease Asn levels by inhibiting WNT/β-catenin signaling, this would thus associate with a better outcome.

### 4.2 Estrogens and estrogen receptors affect asparagine synthesis

The hormone estradiol has been hypothesized to decrease the overall incidence of CRC. Males produce a basal level of estradiol (10–40 pg/ml), which is at a 10-fold lower concentration than in females (Icahn School of Medicine at Mount Sinai, 2021). The protective effects of estradiol are through activation of the nuclear estrogen receptor (ERβ) in the colon mucosal epithelium. Activation of ERβ causes pro-apoptotic signaling, inhibition of inflammatory signals, and modulation of the tumor microenvironment, including inactivation of IL-6 and increased DNA repair capacity (Edvardsson et al., 2011). ERβ expression is significantly reduced in tumor tissues compared to normal tissues, and in advanced stages of colon cancer, ERβ expression can be completely eliminated. ERα is not expressed in the colonic epithelium, therefore understanding the protective role of ERβ is of primary importance in CRC (Caiazza et al., 2015).

Loss of ERβ expression in CRC has been associated with more advanced stages of cancer and poorer prognosis compared to patients with high ERβ expression (Rudolph et al., 2012). In CRC cells, ERβ could upregulate PTEN and downregulate PI3K/AKT/mTOR signaling pathways, thus promoting autophagy (Wei et al., 2019). In Figure 2, we propose that an inverse relationship exists between ERβ and PTEN/PI3K/AKT which modulates ASNS expression and influences CRC progression; PTEN can inhibit PI3K/AKT signaling, and loss of PTEN expression co-occurs with KRAS and PIK3CA mutations (De Roock et al., 2011), enhanced PI3K/AKT signaling can induce ERβ ubiquitination and degradation (Zhou and Liu, 2020). We propose that when ERβ expression is lost, ASNS expression increases, driving Asn production (Figure 2). Activation of PI3K/AKT signaling by mutant *KRAS* can cause ERβ degradation and disables its tumor suppressing effect. When estrogen levels are low, there is decreased activation of ERβ, therefore PTEN can no longer inhibit PI3K/AKT. As activation of PI3K/AKT induces ASNS expression, this cascade causes increased Asn synthesis, which predisposes female patients to a poorer prognosis.

### 4.3 Estrogen regulates tumor microenvironment and indirectly influences Asn bioavailability *via* tuning GPER

The GPER is a membrane estrogen receptor encoded by the *GPER1* gene, which exercises the functions of an estrogen receptor in the absence of nuclear ERs (Gaudet et al., 2015). In CRC, GPER differentially regulates the effects of 17β-estradiol on CRC cell proliferation depending on the tumor microenvironment: 17β-estradiol and a GPER agonist suppressed migration and proliferation in a normoxic tumor microenvironment, while they enhanced migration and proliferation under hypoxia, and these effects were lost in GPER-silenced cells (Bustos et al., 2017). Hypoxia is a decrease in the normal level of tissue oxygen tension (Muz et al., 2015) and typically occurs in advanced cancers (Petrova et al., 2018). Cells respond to hypoxic conditions through hypoxia-inducible transcription factor 1 (HIF-1) which activates the expression of the pro-angiogenic growth factor vascular endothelial growth factor (*VEGF*), a hypoxia-response gene (Harris, 2002). Specifically in CRC, the expression of HIF-1α and VEGFA (an important angiogenesis regulator in VEGF family) were associated with poorer CRC prognosis (Cao et al., 2009). Under normoxia, 17β-estradiol suppressed VEGFA expression, and its expression was lost in GPER-silenced cells, whereas 17β-estradiol increased VEGFA expression (Bustos et al., 2017).

Ataxia telangiectasia mutated (*ATM*) is a key DNA damage repair gene, and loss of ATM expression was associated with adverse prognosis in CRC (Beggs et al., 2012). *ATM* expression was decreased in both hypoxia and normoxia environments *via* GPER signaling (Bustos et al., 2017). These findings suggest a synergistic effect of estrogens and hypoxia in ERβ-negative CRC that represses *ATM* expression to promote cancer cell proliferation and migration, which is induced by hypoxia *via* GPER signaling, activating HIF-1α and VEGFA (Bustos et al., 2017). Furthermore, a sex and stage-specific association has been observed, in which GPER expression was only associated with poor survival in CRC stages III-IV females but not in stage-matched males (Bustos et al., 2017). Lack of ERβ was associated with higher cancer stages of CRC and greater tumor extent (Rudolph et al., 2012). Thus, it might be assumed that GPER could be the primary estrogenic signaling pathway if ERβ expression is low or lost at later stages in females, resulting in poorer clinical outcomes than in earlier stages.

There are links between GPER signaling and downstream targets of KRAS (Chaturantabut et al., 2019; Xu et al., 2020; Shi et al., 2022), wherein activation of GPER increases PI3K/AKT *via* cyclic adenosine monophosphate (cAMP) (Yu et al., 2017; Chuang et al., 2020). Increased expression of genes linked to cAMP was also observed in CRC tumors from female patients (Sun et al., 2020). PI3K/AKT is a downstream target of KRAS which regulates ASNS *via* NRF2 and ATF4. Thus, GPER is linked to ASNS regulation *via* its regulatory effects on PI3K/AKT through cAMP (Figure 2).

Therefore, sex interacts with Asn synthesis and CRC through GPER activation; nutrient supply influences the function of estrogen and GPER1 in *KRAS*-mutant CRC by upregulating ASNS expression, Asn synthesis, PI3K/AKT and HIF-1α-mediated SOX12 overexpression (Figure 2). Due to the multifaceted roles of nuclear receptors in cellular homeostasis, the links between GPER1 signaling and ASNS expression require further mechanistic investigation.

### 4.4 AMPK, asparagine metabolism, and sex differences in right-sided colon cancer

5′AMP-activated protein kinase (AMPK) and mTORC1 play important roles in nutrient signaling and balancing cellular energy homeostasis by sensing cellular ATP and nutrients such as amino acids (Chun and Kim, 2021). We previously discovered downregulation of AMPK alpha subunit (AMPKα) and enriched mTORC1 signaling pathway in females with right-sided colon cancer (RCC) compared to left-sided colon cancer under a nutrient deplete environment (Sun et al., 2020). We also observed enhanced Asn synthesis and amino acids uptake in RCC tissues from females (Cai et al., 2020), which was not observed in RCC tissues from males. Asn and Gln can promote mTORC1 activity (Meng et al., 2020). Moreover, Gln can activate mTORC1 at the surface of the lysosome through the ASNS-mediated production of ATP by inhibiting AMPK (Bodineau et al., 2021). Therefore, enhancement of *de novo* Asn synthesis that upregulates mTORC1 by downregulating AMPK to promote CRC growth is hypothesized in tumors from females with RCC.

### 4.5 Sex could interact with the inhibitory effect of metformin on CRC asparagine synthesis

Diabetes mellitus and CRC have similar deregulation of cellular and molecular pathways in their disease pathogenesis, such as PI3K/AKT/mTOR signaling, WNT/β-catenin pathway, and the AMPK pathway (Yu et al., 2022). Both diseases also have common risk factors including obesity, inflammation, and poor diets (Gonzalez et al., 2017). Metformin, a biguanide compound commonly used for treating diabetes, has shown great potential in CRC treatment (Higurashi and Nakajima, 2018), as suggested by its capability to activate AMPK and inhibit TGF-β/PI3K/AKT signaling transduction (Xiao et al., 2022). Moreover, by functioning as a type of ETC inhibitor that impairs mitochondrial respiration to cause depletion of TCA-cycle-derived Asp, metformin in combination with ASNase or dietary Asn restriction is able to halt tumor growth by limiting *de novo* Asn synthesis (Krall et al., 2021) (Figure 3). Therefore, metformin plays a comprehensive role in Asn metabolism by regulating its upstream substrate Asp, downstream mTORC1 inhibition (Bordini et al., 2017), and AMPK activation which induces p53 phosphorylation and activation (Yi et al., 2019) (Figure 3). Furthermore, compared with *KRAS* wild-type metastatic CRC (mCRC), *KRAS*-mutant mCRC had a greater benefit from metformin administration which inactivated AKT/mTOR signaling and cell proliferation (Xie et al., 2020).

Studies have shown that there are possible sex-related factors that could alter the outcomes of metformin treatment on diabetic CRC. One study using two large cohorts discovered that type 2 diabetes (T2D) was associated with higher CRC risk in males, but not in females (Ma et al., 2018). Diabetic female CRC patients who took metformin had significantly lower CRC-specific mortality than males, showing a sex-specific difference in the effect of metformin on diabetic CRC patients (Park et al., 2017). Such sex differences could be attributable to sex hormone levels, as metformin usage had no significant impact on CRC-specific survival in postmenopausal female CRC patients with diabetes (Cossor et al., 2013). In human endometrial cancer cells, metformin increased ERβ expression and AMPK phosphorylation (Zhang et al., 2017). As estrogens bind to the ERs to exert their functions (Fuentes and Silveyra, 2019), and postmenopausal and diabetic female CRC patients have lower levels of estrogens, it is possible that this underlies the lack of benefit from metformin in those women with CRC (Figure 3). However, the role of metformin with sex interactions in CRC warrants further investigation.

## 5 Discussion

This review has examined and synthesized findings from a vast and rapidly growing body of studies investigating endogenous and exogenous Asn regulators in relation to CRC. Factors that alter intracellular Asn were mainly focused on the proteasome, *ASNS* expression, and the influence of genetic variation, the microenvironment, and other metabolic regulation. We also summarized the extracellular sources of Asn including exposures to Asn *via* dietary intake, microbial metabolism, and these potential effects on cancer progression.

This review has also highlighted a major limitation in current research, in that no study has conducted an evaluation of self-reported dietary exposure to Asn in a population-based study or in smaller clinical series of cancer patients, due to the limited ability to quantify Asn in foods at present. Therefore, unlike many other dietary components, data on the association between CRC risk and prognosis and Asn from the diet are lacking in human studies. However, there is an ongoing clinical trial that assesses nonessential amino acid restricted medical food by measuring biomarkers and tolerability of CRC (ClinicalTrials.gov, 2022). It is possible that such a study will observe an association between nonessential amino acids, including Asn, and CRC outcomes. This also exemplifies the potential of examining dietary patterns that target Asn synthesis through regulating ASNS expression or abundance of its substrates. ASNase is a hydrolase that catalyzes Asn and is widely used in food processing. Specific foods and dietary patterns can result in increased levels of ASNase expressed by various gut microbiota that may reduce susceptibility to CRC. It might be plausible to measure gut ASNase using stool samples and multi-omics analysis and to determine its association with CRC risk and clinical outcomes.

**FIGURE 4:**
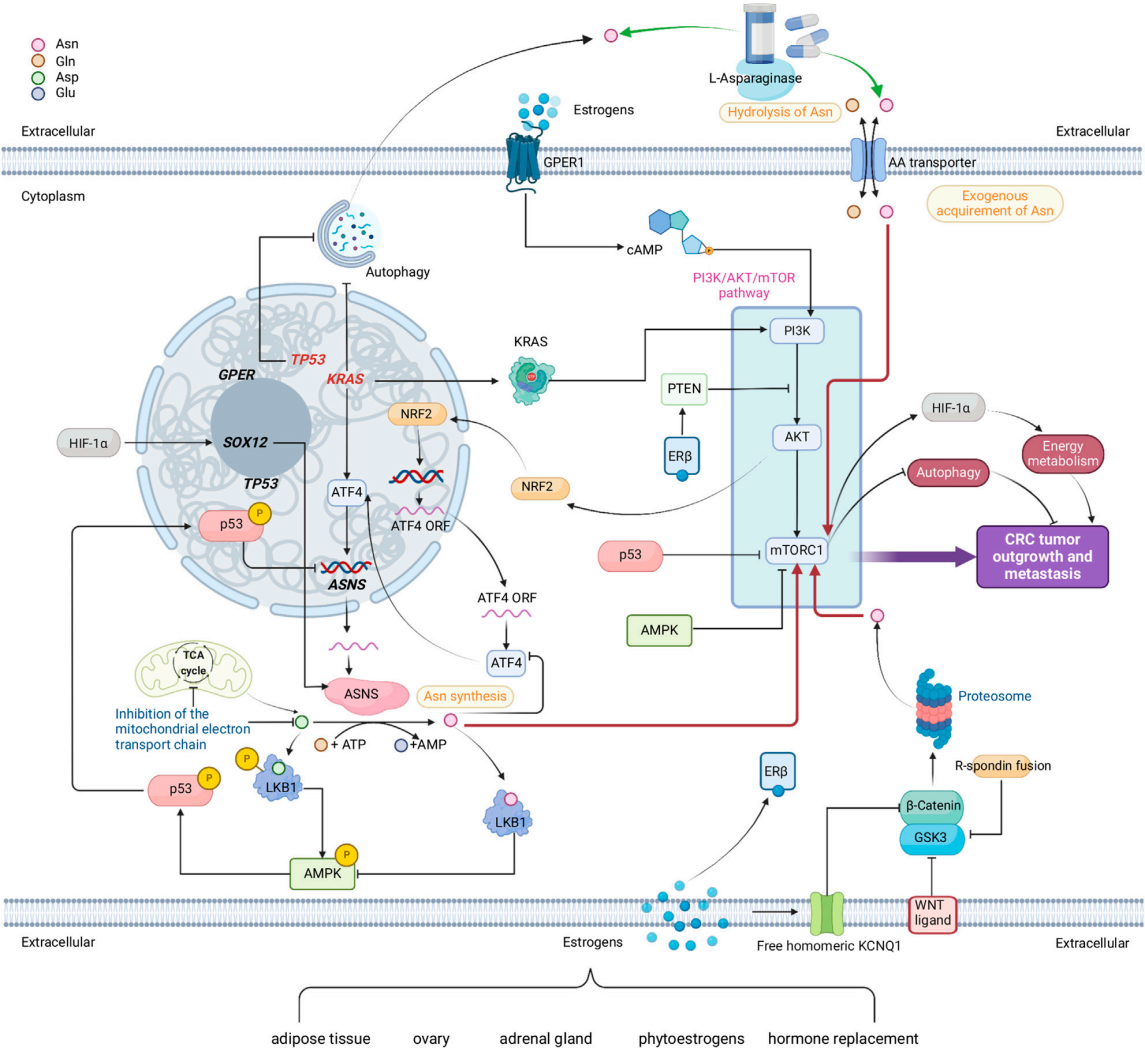
A comprehensive network comprised of multiple metabolic pathways that link to endogenous Asn bioavailability in CRC and the possible interplay of sex. Red arrows show how Asn activates mTORC1. Green arrows show how L-Asparaginase reduces Asn levels. Genes in red are mutated. Sources of estrogens are adipose tissue, ovary, adrenal gland, phytoestrogens, and hormone replacements. Estrogens, nuclear estrogen receptor β (ERβ) and membrane bound G protein coupled estrogen receptor (GPER) demonstrate the potential interplay between sex and Asn bioavailability in CRC. Created with BioRender.com.

The current literature in this area has also led us to hypothesize that sex-specific differences exist in the association between Asn and CRC development and outcomes. We proposed five models of sex interactions (Section 4) that are related to: ion channel activation, ERs, tumor microenvironment, critical pathways with location and sex-specificity, and metformin usage. In addition, sex hormones such as estrogens also contribute to sex specificity in Asn metabolism and CRC. A comprehensive summary of intracellular Asn production, extracellular Asn regulation, and putative sex interplay is displayed in Figure 4.

These hypotheses can potentially explain how sex mediates Asn metabolism in CRC, however, these are not solid causations and should be validated in future experiments and clinical trials. Future research into sexual dimorphism in Asn and CRC could also be aimed at the examination of environmental pollutants. Pollutants such as bisphenols and pesticides activate GPER to mediate estrogenic effects, and consequently influence tumor cell function including proliferation, metastasis, and apoptosis (Qie et al., 2021). Moreover, a thorough holistic assessment of dietary intake of Asn using metabolomics techniques for precision nutrition (Tebani and Bekri, 2019) and adopting subgroup analyses by sex through well-designed clinical trials may inform the sex differences in associations between CRC and dietary exposures to Asn.

## 6 Conclusion

Asn metabolism plays a critical role in CRC tumor proliferation, metastasis, and survival, as shown in both experimental and observational studies where sex-specific associations were seen in population cohorts. Currently, the role of Asn metabolism in CRC is not well understood. The field of environmental health sciences provides a useful tool by enumerating possible exposure pathways that influence Asn bioavailability at both the external environmental exposures level and internal metabolic regulatory aspect. More importantly, in this narrative review, we synthesized these findings to provide several novel hypotheses that tentatively explain the sex-specific phenomena in CRC related to Asn metabolism. We also provide explanations from the perspectives of their biological mechanisms to provide avenues for direction in future studies.

## Permission to reuse and copyright

The Confirmations of Publication and Licensing Rights granted by BioRender (www.biorender.com) for four figures (Figures 1–4) included in this article are attached to the submission materials. Agreement numbers for Figures 1–4 are RI24981UV5, HW24981QAU, DT24981KZW, and TG24981AGE respectively.
